# The Hydrophobic Ligands Entry and Exit from the GPCR Binding Site-SMD and SuMD Simulations

**DOI:** 10.3390/molecules25081930

**Published:** 2020-04-21

**Authors:** Jakub Jakowiecki, Urszula Orzeł, Sathapana Chawananon, Przemysław Miszta, Sławomir Filipek

**Affiliations:** 1 Biological and Chemical Research Centre, Faculty of Chemistry, University of Warsaw, 02-093 Warsaw, Poland; jjakowiecki@chem.uw.edu.pl (J.J.); u.orzel@student.uw.edu.pl (U.O.); sathapana.chawananon@gmail.com (S.C.); pmiszta@chem.uw.edu.pl (P.M.); 2 Department of Chemistry, Faculty of Sciences, Sorbonne Université, 75005 Paris, France

**Keywords:** G protein-coupled receptors, cannabinoid receptors, sphingosine-1-phosphate receptors, lysophosphatidic receptors, steered molecular dynamics, supervised molecular dynamics

## Abstract

Most G protein-coupled receptors that bind the hydrophobic ligands (lipid receptors and steroid receptors) belong to the most populated class A (rhodopsin-like) of these receptors. Typical examples of lipid receptors are: rhodopsin, cannabinoid (CB), sphingosine-1-phosphate (S1P) and lysophosphatidic (LPA) receptors. The hydrophobic ligands access the receptor binding site from the lipid bilayer not only because of their low solubility in water but also because of a large N-terminal domain plug preventing access to the orthosteric binding site from the extracellular milieu. In order to identify the most probable ligand exit pathway from lipid receptors CB1, S1P1 and LPA1 orthosteric binding sites we performed at least three repeats of steered molecular dynamics simulations in which ligands were pulled in various directions. For specific ligands being agonists, the supervised molecular dynamics approach was used to simulate the ligand entry events to the inactive receptor structures. For all investigated receptors the ligand entry/exit pathway goes through the gate between transmembrane helices TM1 and TM7, however, in some cases it combined with a direction toward water milieu.

## 1. Introduction

G protein-coupled receptors (GPCRs) are membrane proteins that allow cells to recognize diverse extracellular stimuli (like neurotransmitters or hormones) and transduce the signals across the plasma membrane to activate G proteins that pass the signal down the cell. GPCRs are the pharmacological targets for 30–50% of currently used drugs because they are present in signaling routes associated with various diseases such as metabolic diseases, immunological diseases, viral infections, mental dysfunctions, cardiovascular diseases, cancer and inflammatory processes. Most GPCRs bind water soluble ligands. The ligand binding site of the most populated rhodopsin-like (class A) GPCRs is located at the extracellular part of those receptors. The water soluble ligand molecules can easily enter the receptor’s entry hall shortly after being released into the synaptic space since the orthosteric ligand binding site is wide open or semi-open. However, not all GPCR ligands are water soluble and this has some implications on the construction of the ligand binding site and the ligand entering pathway. The two main groups of receptors binding hydrophobic ligands are lipid receptors and steroid receptors and they all belong to class A GPCRs. In this study we focus on the lipid receptors since no crystal structure of the steroid GPCRs is available yet. Typical examples of lipid receptors are: rhodopsin, cannabinoid (CB), sphingosine-1-phosphate (S1P) and lysophosphatidic (LPA) receptors. They are closely evolutionary related to one another displaying a 25%–51% sequence similarity. The highest sequence similarity between different lipid receptor types (51%) exists for S1P_1_ and LPA_1_ receptors by the GPCRdb web service [1]. Although most GPCR ligands access the receptor binding site from water solution, such a scenario is unlikely for lipid receptors because most of their ligands are not soluble in water. Therefore, the hydrophobic ligands attain access to the appropriate receptor via the lipid bilayer. Additionally, some endogenous hydrophobic ligands of lipid receptors (e.g., anandamide and sphingosine-1-phosphate) are synthesized on demand from the membrane components [2,3] and when no longer needed they are metabolized by enzymes capable of penetrating the membrane, such as fatty acid amide hydrolase (FAAH) [4]. Hence, the whole signaling process mediated by lipid receptors can function fast and efficiently without their ligands ever leaving the membrane environment.

**Figure 1:**
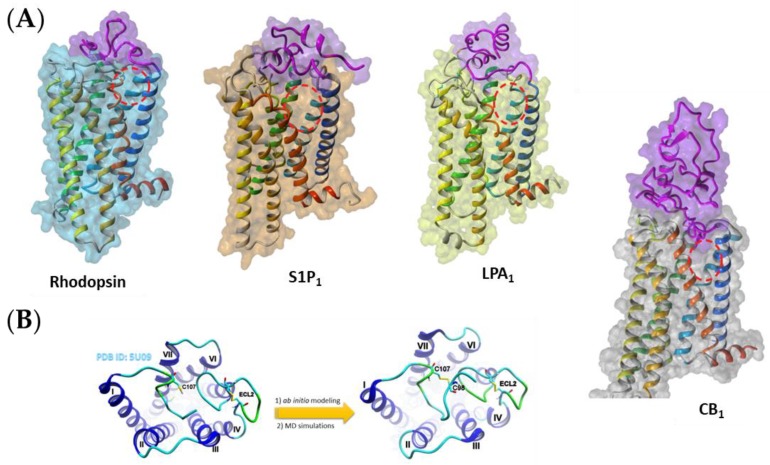
(**A**) The examples of hydrophobic ligand-binding G protein-coupled receptors (GPCRs). The N-terminal domain (purple) prevents the ligand access from the extracellular side. Instead, the possible entrance to the ligand binding site is located between helices TM1 and TM7 (red dashed ovals). (**B**) Modeling of a few residues in the N-terminal part of CB_1_ containing important disulfide bridge. For comparison, on the right, one of the probable conformations of the whole N-terminus of CB_1_ is shown.

Despite the fact that lipid receptors are closely related with other GPCRs from class A, they display one major structural difference compared to other members of that class: they lack the opening at the extracellular side. A large N-terminal domain forms a plug preventing access to the orthosteric binding site from the extracellular milieu (Figure 1). Instead, lipid receptors exhibit a vast crevice between transmembrane helices TM1 and TM7 making the binding site accessible from the membrane. It has been shown that the ligand binding channel is located between those helices in the opsin [5] and CB_1_ cannabinoid receptors [6]. In our current study we investigated the ligand entrance pathway for two other types of lipid receptors: lysophosphatidic acid receptor type 1 (LPA_1_) and sphingosine-1-phosphate receptor type 1 (S1P_1_), as well as verified the results previously obtained for the CB_1_ cannabinoid receptor homology model. This time the initial coordinates for all molecular dynamics (MD) simulations were based on the crystal structures of the appropriate receptors. For CB_1_ we also modeled the whole N-terminal part and we have simulated the entrance of two agonists, anandamide and Δ^9^-THC, into the structure of CB_1_. For the S1P_1_ receptor, the SMD simulations of ligand pulling were performed for two agonists of that receptor, sphingosine-1-phosphate (S1P) and fingolimod-(*S*)-phosphate ((*S*)-FTY720-P [7]). For the LPA_1_ receptor, the similar SMD simulations were performed for lysophosphatidic acid, which is the endogenous agonist of that receptor. A verification of the TM1–TM7 ligand channeling pathway was done for selected ligands using the supervised molecular dynamics (SuMD) method.

## 2. Results and Discussion

### 2.1. Steered Molecular Dynamics (SMD) Simulations

**Figure 2:**
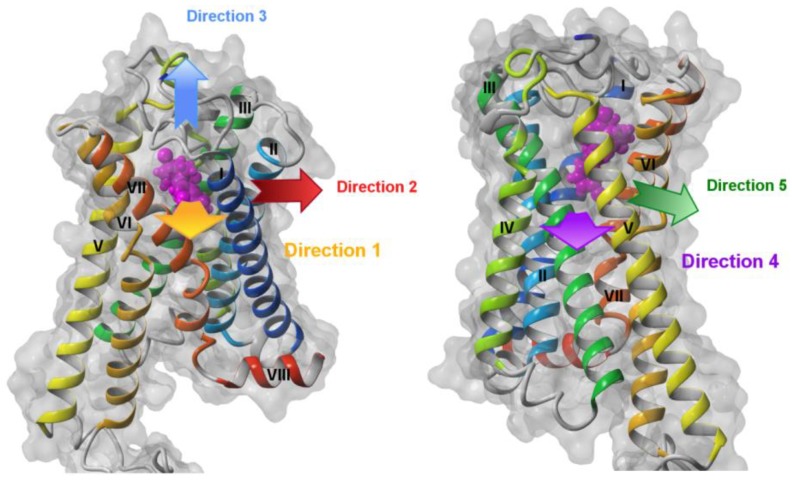
Directions of ligand pulling in SMD simulations. Direction 1 (yellow arrow): between transmembrane helices TM1 (blue) and TM7 (orange-red), Direction 2 (red arrow): between TM1 (blue) and TM2 (cyan), Direction 3 (blue arrow): “up” towards the extracellular side, Direction 4 (purple arrow): between TM4 (yellow-green) and TM5 (yellow), Direction 5 (green arrow): between TM5 (yellow) and TM6 (orange). The structure of the CB_1_ receptor without the whole N-terminus is shown but with modeled residues 96–99 containing residues participating in ligand binding in the orthosteric site.

The SMD simulations of ligand pulling from the orthosteric site were performed for the most probable directions where the crevices between transmembrane helices were the widest and also for the additional direction towards the extracellular side (Figure 2). To obtain the statistically valid results at least three repeats of SMD simulation were performed for each pulling direction. Ligands were pulled out using those ligand atoms that were located closest to the selected direction of pulling—it means that they were different for each direction. This strategy prevented making a rotation of the ligand in the binding site, which could possibly jam that direction. Most of the receptor structures were inactive structures (crystallized with antagonists or inverse agonists) and only one structure (CB_1_ receptor PDB ID: 5XR8) was active.

#### 2.1.1. SMD Simulations of CB_1_ Complexes with Two Antagonists and One Agonist

**Figure 3:**
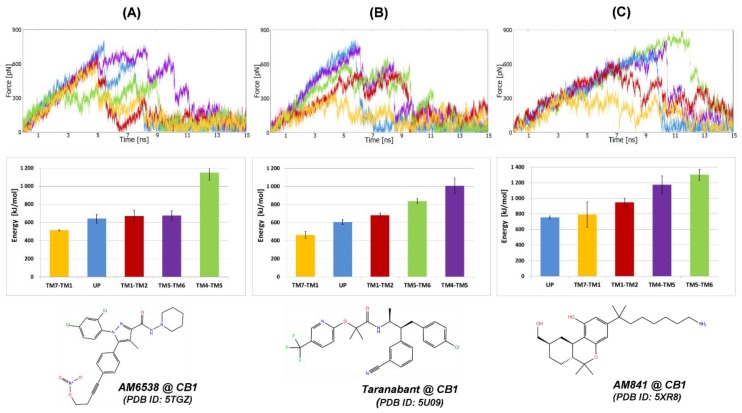
SMD simulations of three orthosteric ligands pulling from different CB1 receptor crystal structures; (**A**) AM6538 (PDB ID: 5TGZ, resname: ZDG); (**B**) taranabant (PDB ID: 5U09, resname: 7DY); (**C**) AM841 (PDB ID: 5XR8, resname: 8D0). Top row: exemplary force-time profiles for each pulling direction of the particular ligand; middle row: energy required to pull out the ligand from the binding site; bottom row: structures of the ligands. Colors denote the particular direction of pulling according to Figure 2.

For CB1 receptor complexes with antagonists (AM6538 and taranabant), the smallest work (energy) required to pull the ligand out of the binding site was recorded for direction between TM1 and TM7 (Figure 3A,B). For the third ligand, agonist AM841, the smallest work was recorded for direction UP to the extracellular site, however, statistically this direction was equivalent to the direction between helices TM1 and TM7 (Figure 3C). The smallest energy (Figure 3, middle panels), calculated by numerical integration of the force-distance data, was associated with the smallest maximal force for the same direction of pulling (Figure 3, top panels). It means that this direction not only requires the smallest work but also there are the smallest forces on the way between the ligand binding site and the outside of the receptor. For the CB_1_ receptor, we have not used the whole modeled N-terminus (Figure 1A) but only its fragment (residues 96–99) that is present but not visible in the crystal (Figure 1B). This fragment contains the important disulfide bond (C98–C107) and the residues from this fragment (particularly F102 and M103) participate in the ligand binding in the orthosteric site. So even without the completely reconstructed N-terminal part, which could additionally block the ligand exit to the water milieu, the direction between TM1 and TM7 towards the membrane milieu was the most favorable.

#### 2.1.2. SMD Simulations of S1P_1_ Complexes with Two Agonists

**Figure 4:**
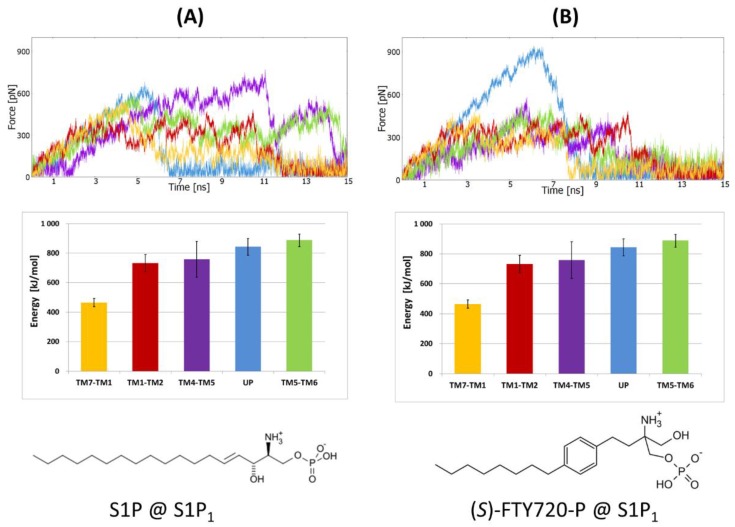
SMD simulations of two orthosteric agonists pulling from the same S1P_1_ receptor crystal structure (PDB ID: 3V2Y); (**A**) sphingosine-1-phosphate (S1P) ligand; (**B**) (*S*)-FTY720-P ligand. The panel layout and the meaning of colors the same as in Figure 3.

For both ligands of the S1P_1_ receptor the same results were obtained as for the CB_1_ receptor (Figure 4). The direction between helices TM1 and TM7 proved to be the most accessible for the ligands. This direction required the smallest work (equivalent with direction UP for S1P ligand) and was characterized by the smallest maximal force (the smallest friction) on the way out. In both cases, the ligands moving between TM1 and TM7 were pulled by their phosphate group suggesting that they are entering the receptor with their hydrophobic tails first. As described in the methods, the atoms used for pulling are different for each tested direction to not generate rotation of the ligand in the binding site and jamming the exit. For the direction UP for (*S*)-FTY720-P, the ligand collided with part of the N-terminus when leaving the receptor. This collision caused a significant deformation of the N-terminal domain structure, and therefore a sharp peak of the friction force was recorded (Figure 4B).

#### 2.1.3. SMD Simulations of LPA_1_ Complex with Endogenous Agonist

**Figure 5:**
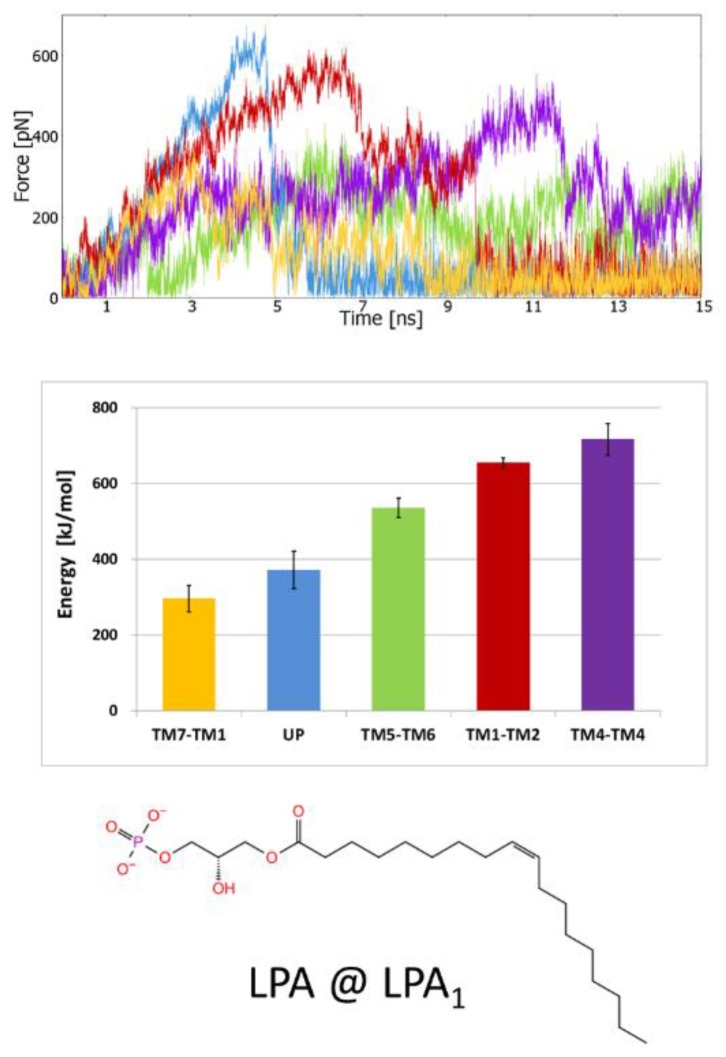
SMD simulations of the orthosteric ligand (*S*)-lysophosphatidic acid ((*S*)-LPA) pulling from LPA_1_ receptor crystal structure (PDB ID: 4Z34). The panel layout and the meaning of the colors are the same as in Figure 3.

For the LPA_1_ receptor with docked endogenous LPA agonist (we used (*S*)-LPA, although both enantiomers have similar activity [8]) we conducted a set of SMD simulations and obtained the same results as for the above two lipid receptors. The smallest work required to leave the receptor and also the smallest friction (calculated as the smallest maximal force) were obtained for the TM1–TM7 direction (Figure 5). As for the S1P_1_ receptor ligands, LPA was pulled out by pulling the phosphate group (for directions TM1–TM7, TM1–TM2 and UP) which suggests that the entrance of the ligand into the orthosteric binding site of the LPA_1_ receptor from the membrane environment is performed in ‘hydrophobic tail first’ orientation. For directions TM4–TM5 and TM5–TM6 the ligand was pulled by the terminal carbon atom to prevent the rotation of the ligand in the binding site and jamming the exit. In nearly all investigated systems a pulling direction TM1–TM7 was preferable, while the next probable direction was UP suggesting that in reality both directions can be combined and the ligands can partly explore the water milieu while exiting/entering the receptor bindings site.

### 2.2. The Supervised Molecular Dynamics (SuMD) Simulations of Ligand Entrance

The conducted SMD simulations indicated that crossing through the TM1–TM7 crevice is the preferable way to exit the orthosteric binding site of lipid receptors. Since the steric barriers were the lowest along that pathway, we assume it is also a preferable route of ligand entrance. Such a scenario was subsequently checked by us using SuMD simulations. In this method, no pulling force is employed but instead, the normal all-atom MD simulation is restarted from the latest checkpoint when a distance of a ligand to the center of a binding site increases too much. We used only the agonists entering the inactive structures of the lipid receptors to study how the ligand can go through the TM1–TM7 gate but also whether the inactive receptor structure is ready enough to accept the agonist that will be changing the receptor structure.

#### 2.2.1. SuMD Simulations of Two Agonists Entering Inactive CB_1_ Receptor Structure

**Figure 6:**
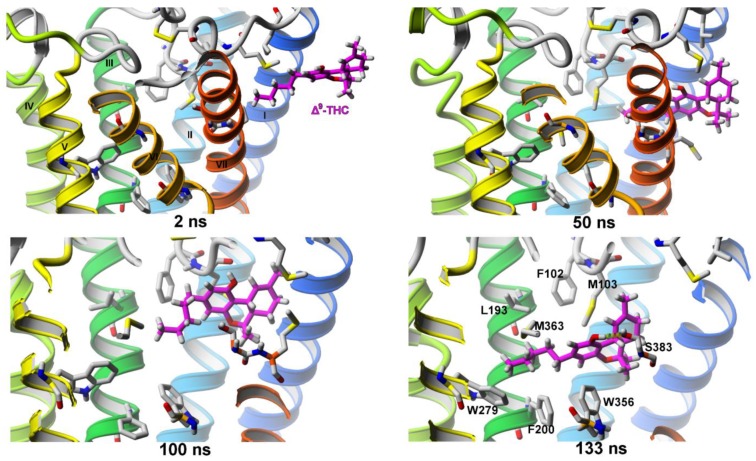
SuMD simulation of Δ^9^-THC entrance into the orthosteric binding site of the CB_1_ cannabinoid receptor.

**Figure 7:**
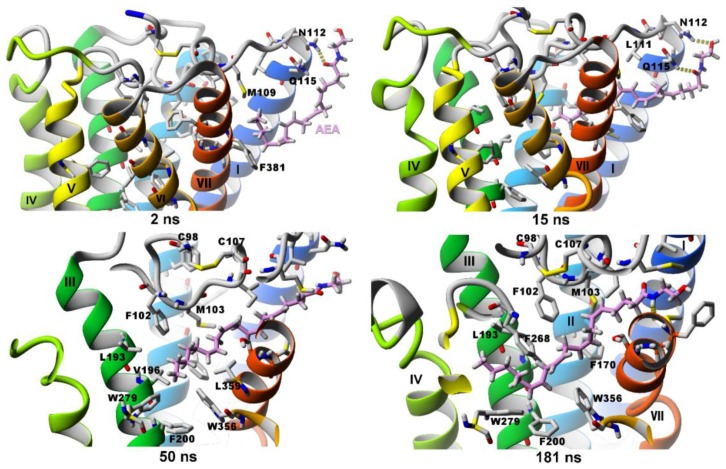
SuMD simulation of anandamide entrance into the orthosteric binding site of the CB_1_ cannabinoid receptor.

For the supervised molecular dynamics (SuMD) simulations of ligand entrance into the orthosteric binding site of the CB_1_ cannabinoid receptor, we used a refined crystal structure (PDB ID: 5U09) of that receptor, with four N-terminal residues (I96, Q97, C98 and G99) added and a C98–C107 disulfide bond recreated. The remodeled fragment (called by us EC0—extracellular loop 0) is essential for the ligand binding process, since some of the residue sidechains (F102 and M103) are reaching deep into the orthosteric binding site to form van der Waals interactions with orthosterically bound ligands (Figure 6 and Figure 7), while the other residues of this region are cushioning the surface of the ligand binding channel. We tested both ends of the ligands (hydrophilic and hydrophobic) to enter the receptor, as we have done for the same agonists, Δ^9^-THC and anandamide, in our simulations of a homology model of CB_1_ [6].

##### Δ^9^-THC Entrance to CB_1_ Receptor

The Δ^9^-THC molecule moves into the ligand binding channel, located between TM7–TM1, directly from the membrane and only in the “alkyl chain first” orientation (Figure 6, 2 ns and 50 ns). After ~85 ns of SuMD simulation, it reaches a metastable state between TM7 and EC0 and it remains there for another 30–35 ns (Figure 6, 100 ns). After that time the THC molecule goes deeper into the receptor to reach the orthosteric binding site, where it is stabilized by mostly hydrophobic interactions as well as a H-bond formed between the ligand’s hydroxyl group and the residue S383^7.39^ (Figure 6, 133 ns). In addition, we have observed some rearrangement of the transmission switch (residues F200^3.36^ and W356^6.48^) [9] after the ligand molecule got near those residues. That rearrangement is believed to be the first indicator of receptor activation [10,11].

##### Anandamide (AEA) Entrance to the CB_1_ Receptor

Anandamide enters into the ligand binding channel of the CB1 receptor in a manner similar to Δ^9^-THC: (i) it enters the crevice between TM1–TM7 with only “alkyl chain first” orientation, (ii) the first stage of ligand entrance occurs spontaneously even when not using the supervision algorithm. The formation of H-bonds between the anandamide polar head and residues N112 and Q115 promotes the ligand orientation required for the entrance (Figure 7, 2 and 15 ns). After 50 ns of SuMD simulation, a part of the anandamide molecule reaches the orthosteric binding site and forms first contacts with residue W356^6.48^ leading to the first signs of a transition switch rearrangement (Figure 7, 50 ns). The pose, which we believe is a correct AEA binding pose, was reached after 181 ns of SuMD simulation. In this pose, there are π–π interactions formed between double bonds of AEA and residues F170^2.57^, F200^3.36^ and W356^6.48^ (Figure 7, 181 ns).

#### 2.2.2. Sphingosine-1-phosphate (S1P) and (*S*)-FTY720-P Entrance into the S1P_1_ Receptor

**Figure 8:**
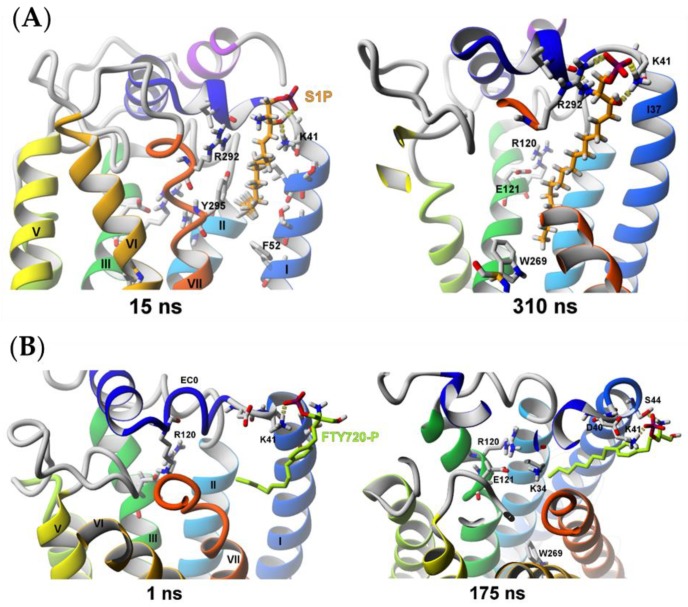
SuMD simulation of two agonists trying to enter to the inactive S1P_1_ receptor structure (PDB ID: 3V2Y). (**A**) S1P ligand; (**B**) (*S*)-FTY720-P ligand.

We performed altogether nine SuMD simulations for S1P entrance and four for (*S*)-FTY720-P entrance using different protonation forms of the ligands and different entrance orientations. Both S1P and (*S*)-FTY720-P entered the S1P_1_ receptor only partially, never reaching their final binding site found in the crystal structure of the S1P_1_ receptor with the similar in structure antagonist (being a sphingolipid mimic) which is characterized by binding of the ligand’s phosphate group to the residues K34^NT^ and R120^3.28^. The lack of full entrance might have been caused by the strong ionic interactions of the phosphate group of those ligands formed with the residues K41^NT^ and R292^7.34^ located near the entrance to the channel leading to the orthosteric ligand binding site (Figure 8A). Nevertheless, the long hydrophobic tail of S1P is reaching the transmission switch (W269^6.48^), so probably after the action of the switch and making more room in the receptor interior the deeper entrance of the agonist will be possible (Figure 8A, 310 ns). The (*S*)-FTY720-P agonist remained half outside of the receptor bound to residues K41^NT^ and S44^1.31^. This ligand is bulkier than S1P so it has to overcome higher steric barriers and this is why the 175 ns probably was not enough time to simulate the ligand entrance (Figure 8B, 175 ns), compared with the time required for S1P for its partial entrance (Figure 8A, 310 ns).

#### 2.2.3. SuMD Simulations of LPA Ligand Entrance into the LPA_1_ Receptor

**Figure 9:**
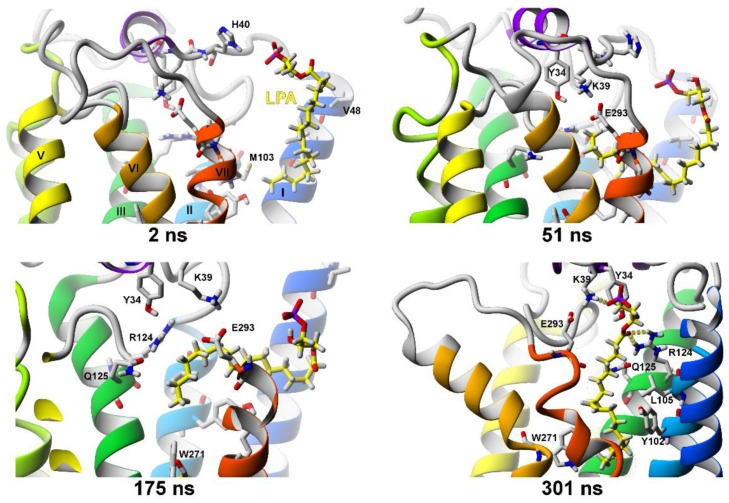
SuMD simulation of LPA entrance into the inactive LPA_1_ receptor structure (PDB ID: 4Z34). After 175 ns the SuMD was turned off and a regular MD was carried out.

We performed four SuMD simulations of LPA entrance into the LPA_1_ receptor. In two of them, the “alkyl chain first” ligand entrance orientation was set, and in both simulations the whole ligand molecule entered the receptor, although the two simulations yielded slightly different ligand binding modes. In the ligand pose obtained from simulation 1 (143 ns SuMD + 41 ns MD), the LPA phosphate group formed ionic interactions with residues K39^NT^ and R124^3.28^ (and in some frames a hydrogen bond with Y34^NT^) while the other end of the molecule formed a contact with residue W271^6.48^ involved in a transmission switch (not shown). In the pose from simulation 2 (175 ns SuMD + 33 ns MD) the phosphate group of the ligand formed an ionic interaction with the residue K39^NT^ while the carbonyl group of the ligand formed an H-bond with R124^3.28^ (Figure 9, 208 ns). At the same time, the opposite end of the molecule formed the hydrophobic interactions with the residue W271^6.48^ (from the transmission switch). After 175 ns the SuMD was turned off and regular molecular dynamics (MD) was carried out so no supervision was needed for the ligand to move into the binding site from that state. This indicated that the entrance of this flexible ligand was pretty smooth and the long-distance electrostatic attraction between the phosphate group of ligand to residues in the binding site was guiding the ligand. In simulations 3 and 4, a possibility of the “polar head-first” entrance orientation was explored but, in both cases, the ligand entered only partially.

#### 2.2.4. Comparison of SuMD Simulations to Earlier Data

In our previous paper on SMD/SuMD of the CB_1_ receptor [6], we used the homology model of the inactive CB1 receptor built with two crystal structures as templates: S1P_1_ receptor (PDB ID: 3V2Y) and LPA_1_ receptor (PDB ID: 4Z34). We simulated the exit/entrance of Δ^9^-THC and anandamide entrance into the CB_1_ receptor homology model and we concluded that residues F174 and F177 are involved in a gating mechanism, allowing only some ligands to go in while rejecting the others. In contrast to what we had observed that time, during current SuMD simulations based on CB_1_ the crystal structures that we observed had no gating mechanism. Therefore, the first stage of the ligand (Δ^9^-THC or AEA) entrance into the CB_1_ receptor occurs spontaneously even with the supervision algorithm. The ligand situated near the entrance to the ligand binding channel between TM1 and TM7, assuming its orientation is correct, will diffuse into the channel without a need to overcome any steric barriers. Instead, the ligand is stimulated to enter the receptor by the hydrophobic and highly flexible sidechains of residues surrounding the entrance to the binding channel, such as: M109, L111, F381 and M384 (Figure 6 and Figure 7). At the same time, the residues F174 and F177 that we previously thought to be engaged in the gating mechanism are not located in the orthosteric ligand binding channel but rather in the pocket between helices TM1 and TM2 where the allosteric modulators bind.

The ligand entrance pathway between TM1 and TM7 has also been suggested for the S1P_1_ receptor based on its crystal structure [12] and for rhodopsin [5]. Another pathway, between helices TM6 and TM7, was suggested for 2-arachidonoylglycerol (2-AG) entering the cannabinoid CB_2_ receptor homology model based on rhodopsin. The ligand entered the CB_2_ receptor, with its polar head entering first, and during nearly 2 μs of MD simulation 2-AG was not able to enter the binding site while most of its hydrophobic tail stayed outside [13]. For the LPA_1_ receptor, it was proposed that the most probable ligand entrance is from the extracellular milieu [14]. It was based on the analysis of the crystal structure of the LPA_1_ receptor and 100 ns MD simulations of the receptor without a ligand to monitor a distance between helices TM1 and TM7. In a recent publication [15], the hypothetical directions of ligand entry into the orthosteric binding sites of lipid receptors are discussed based on their crystal structures. Both vertical (from water) and lateral (from the membrane, most likely between helices TM1 and TM7) directions are suggested. However, taking into account that N-termini were not reconstructed, the probability of a vertical direction is much smaller. Our simulations were conducted with particular ligands, agonists and antagonists, exiting/entering the orthosteric binding site and the proposed mechanism permits combining a direction between TM1 and TM7 helices for the hydrophobic part of a ligand, while its hydrophilic head contacts the water milieu.

#### 2.2.5. The Energy Profiles and Distances for SuMD Simulations

**Figure 10:**
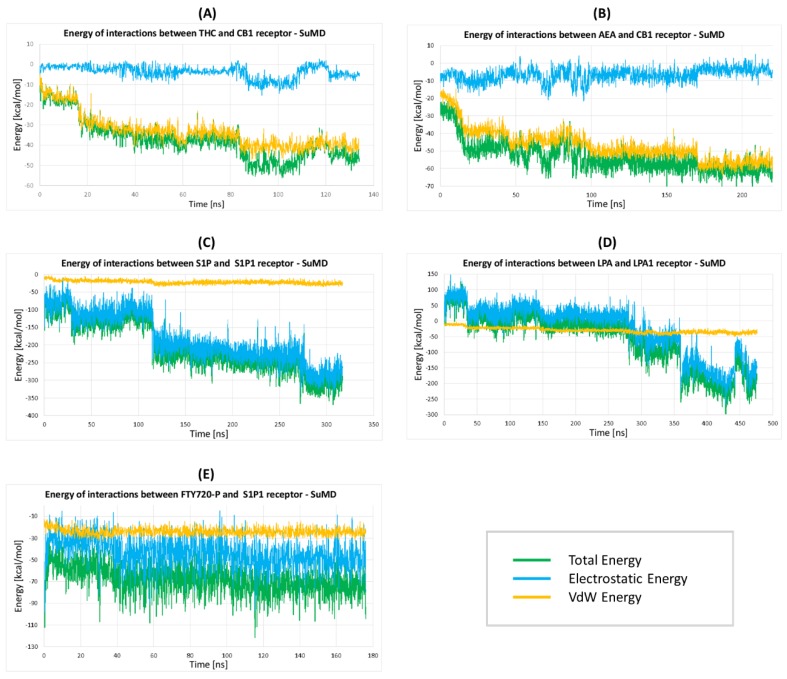
The energy profiles during the ligand approaching the receptor interior in SuMD simulations. (**A**) THC entering the CB1 receptor; (**B**) AEA entering the CB1 receptor; (**C**) S1P entering the S1P_1_ receptor; (**D**) LPA entering the LPA_1_ receptor; (**E**) ((*S*)-FTY720-P entering the S1P_1_ receptor. This ligand only partially entered the receptor. The receptor-ligand interaction energy (green) is divided into electrostatic (blue) and van der Walls (orange) contributions.

We also performed calculations of energy profiles during all SuMD simulations (Figure 10). For hydrophobic ligands THC and AEA (Figure 10A,B) the electrostatic contribution of ligand-receptor interaction energy is close to zero, while the van der Waals energy goes down to about –40 kcal/mol and –56 kcal/mol, respectively, making such binding strong. The entropy contribution is not regarded but since water is removed from the hydrophobic interior of the receptor the ligand binding is even stronger. For S1P and LPA ligands entering their receptors (Figure 10C,D), the van der Waals energy also contributes to the ligand binding: –25 kcal/mol for S1P and –36 kcal/mol for LPA. Both ligands have long hydrophobic tails but also positively and negatively charged groups which contribute to a large decrease in interaction energy resulting in strong binding to the receptor.

Interestingly, in the case of ligand LPA, the electrostatic energy was positive during the initial stages of the ligand entering the receptor so the charged part of the ligand was repelled (Figure 10D) and only van der Waals forces were responsible for attracting LPA into the receptor since the ligand was entering the receptor with its hydrophobic tail first (Figure 9). The (*S*)-FTY720-P only partially entered the S1P_1_ receptor but the obtained van der Walls energy was nearly the same as for the LPA ligand (–25 kcal/mol) indicating good binding of the hydrophobic part of this ligand. Possibly, a much longer time is needed for (*S*)-FTY720-P to enter the receptor binding cavity.

For SuMD simulations, we also estimated how far the simulated ligand entered the receptor and, at the same time, how close it is from the putative center of the binding site. Unfortunately, there are no available crystal structures of the ligand-receptor complexes that we used in our SuMD simulations. Docked ligand poses can be imprecise, therefore, instead of RMSD (root mean square distance) between the SuMD final position and a docked position of the ligand we calculated a distance between the centers of mass of the crystal ligand in its ligand-receptor crystal structure and our ligand in the SuMD final position. To obtain statistically valid results we performed the above calculations for different crystal structures of the same receptor (Table 1).

The average distance between ligand THC in the SuMD final position and the crystal ligands is 3.4 Å on average, while for AEA ligand it is 3.1 Å. It indicates that these ligands are close to their putative final positions inside the CB_1_ receptor. For comparison, a distance between ligands in two different crystal structures of CB1, 8D0 (PDB id: 5xr8) and 7DY (PDB id: 5u09), is a larger value, 5.3 Å, indicating that the ligand poses obtained from SuMD are inside the binding site. Similar results were obtained for LPA_1_ receptor, where the average distance between the crystallized ligands and the ligand poses obtained from SuMD was 3.5 Å. However, for the S1P_1_ receptor ligand, S1P, the distance is much larger (arround 8.0 Å), which means that only the initial binding stages were completed. Another S1P_1_ receptor ligand, (*S*)-FTY720-P, did not enter the orthosteric binding site at all and therefore it was not included in Table 1. Definitely, entering the S1P_1_ receptor is more difficult and requires more time than for the other analyzed lipid receptors.

## 3. Materials and Methods

### 3.1. Obtaining the Receptor Structures

In order to identify the most probable ligand entrance pathway into CB_1_, S1P_1_ and LPA_1_ receptors orthosteric binding sites, we performed at least three repeats of steered molecular dynamics (SMD) and supervised molecular dynamics (SuMD) simulations using various ligands. All receptor structures used for the simulations had been obtained from appropriate crystal structures of human receptors for CB_1_ cannabinoid receptor (PDB IDs: 5TGZ [16], 5U09 [17] and 5XR8 [18]), for the S1P_1_ receptor (PDB ID: 3V2Y [12]), and for the LPA_1_ receptor (PDB ID: 4Z34 [14]). All extracellular loops are present in the crystal structures of all investigated receptors without any missing fragments. Also, these receptor structures contain essential fragments of N-termini. Only in the case of the CB1 receptor did we add a few residues to the N-terminus that can participate in ligand binding. Before docking, we had converted the receptor to the wild type and reconstructed the missing fragments i.e., intracellular loop IC3 and the N-terminus (in case of CB1 it was a fragment of the N-terminus, which is essential for ligand binding). Then, we performed all-atom simulations (100–250 ns depending on the length of missing fragments added) of the receptor before the ligands were docked.

For CB_1_ receptor, the fragment of the N-terminus (residues 96–99, lacking in all available experimental structures) was rebuilt to obtain the important disulfide bond (C98–C107). Residues 96–100 and 107–108 were sampled in Rosetta, using the condition that the C98–C107 disulfide bond must be maintained. Residues from the rebuilt fragment participate in ligand binding in the orthosteric site so they are important for our calculations. For two other receptors, S1P_1_ and LPA_1,_ their lacking parts in the N-terminus were small (15 residues for S1P_1_ and 19 residues for LPA_1_) and they were rebuilt in whole using Rosetta-Membrane [19] employing an ab initio approach (1000 models of each N-termini were generated and scored). Smaller fragments lacking in the crystal structure were refined using the YASARA v.19.9.25 program [20]. For labeling the residues, the classical (Ballesteros–Weinstein) numbering scheme [21] was employed.

### 3.2. Ligand Docking and Preparations for SMD and SuMD Simulations

For the CB_1_ cannabinoid receptor, the SMD simulations of ligand pulling from the orthosteric binding site were performed for ligands found in crystal structures of that receptor: with antagonist AM6538 (PDB ID: 5TGZ, resolution 2.8 Å), with antagonist taranabant (PDB ID: 5U09, resolution 2.6 Å), and with agonist AM841 (PDB ID: 5XR8, resolution 2.95 Å). For the S1P_1_ receptor, the SMD simulations of ligand pulling were performed for two agonists of that receptor, sphingosine-1-phosphate (S1P) and fingolimod-(*S*)-phosphate ((*S*)-FTY720-P), which were docked to the orthosteric binding site of the S1P_1_ receptor crystal structure (PDB ID: 3V2Y, resolution 2.8 Å) instead of antagonist sphingolipid mimic present in the crystal structure. For the LPA_1_ receptor, the similar SMD simulations were performed for the endogenous orthosteric agonist of that receptor, lysophosphatidic acid, docked to the orthosteric binding site of the LPA_1_ crystal structure (PDB ID: 4Z34, resolution 3.0 Å) instead of antagonist ONO9780307, which is present in the crystal structure. To build ligands and perform docking, the Maestro and Glide programs from the Schrödinger Suite release 2019–1 were employed. In all docking experiments we used Glide, also from the Schrödinger suite, using flexible ligands and a rigid receptor grid, which is a default method in Glide. We employed standard precision (SP) for Glide docking and for each ligand we obtained 20–30 poses. A 3D-grid was generated around all orthosteric ligands in the crystal structures of lipid receptors. The binding site was defined as a centroid of orthosteric crystal ligands (in most cases the grid diameter was 20 Å). The default parameters (van der Waals radii scaling factor = 1.0 with partial charge cutoff = 0.25) were used in receptor grid generation. Docking default parameters were used: van der Waals radii scaling factor = 0.80; partial charge cutoff = 0.15; flexible ligand sampling; force field = OPLS3e with a distance-dependent dielectric model.

The quantum-mechanical charges for molecular dynamics were calculated using Jaguar (Schrödinger suite) with functional B3LYP and 6-311G** basis set. The parameter files for ligands were obtained using the ParamChem server employing CGenFF (CHARMM General Force Field) for small molecules [22]. All SMD and SuMD simulations were performed using the NAMD v.2.11 simulation package with CUDA support and a CHARMM36 force field. The CHARMM-GUI service (http://www.charmm-gui.org/) [23,24,25] was used for NAMD input files generation. All receptor structures containing appropriate ligands were placed in a hydrated 1-palmitoyl-2-oleoyl-3-phosphocholine (POPC) bilayer containing 20% (*n*/*n*) of cholesterol and in every case, a short equilibration about 1 ns was carried out prior to the main simulation. 

### 3.3. The SMD and SuMD Simulations

The SMD simulations of ligand pulling from the binding site of all mentioned receptor structures were performed employing five different directions of pulling: (1) between TM1 and TM7; (2) between TM and TM2; (3) towards the extracellular side; (4) between TM4 and TM5; (5) between helices TM5 and TM6 (Figure 2). For all SMD simulations a constant velocity (v = 0.3 m/s) and a virtual spring with force constant k = 70 pN/Å were applied. Ligands were pulled using those atoms that were located close to the selected direction of pulling to avoid rotation and blocking the ligand in the binding site. For each pulling direction, at least three repeats of SMD simulation were performed and each SMD simulation was 15 ns long. The force values were recorded during each of the simulations and were plotted afterward. The energy (work) required for each ligand exit along each pathway was calculated by numerical integration of the force-distance data (Equation).

$$\begin{array}{cc} W=\int \vec{F}\times d\vec{r}= & \int^{\;}\left(F_{x}dx+F_{y}dy+F_{z}dz\right)\\ & \approx \overset{n}{\underset{0}{\sum }}F_{x}\times \Delta x+F_{y}\times \Delta y+F_{z}\times \Delta z\\ & =\overset{n}{\underset{i=0}{\sum }}\frac{\left(F_{x}\left(i\right)+F_{x}\left(i+1\right)\right)}{2}\times \left(x_{i+1}-x_{i}\right)+\frac{\left(F_{y}\left(i\right)+F_{y}\left(i+1\right)\right)}{2}\\ & \times \left(y_{i+1}-y_{i}\right)+\frac{\left(F_{z}\left(i\right)+F_{z}\left(i+1\right)\right)}{2}\times \left(z_{i+1}-z_{i}\right) \end{array}$$

Additionally, we employed the modified supervised molecular dynamics (SuMD) [26] method to simulate the ligand binding events for the lipid receptors. This is a special supervision tabu-like algorithm, monitoring the distance between the ligand and the receptor binding site, which can restart simulation from the checkpoint if that distance increases too much. All details of the SuMD algorithm that were used are described in our previous paper on the CB_1_ receptor [6]. For SuMD simulations, we used only agonists to enter the inactive structures of lipid receptors: anandamide and Δ^9^-THC for the CB_1_ receptor (refined crystal structure PDB ID: 5U09), LPA for the LPA_1_ receptor (refined crystal structure PDB ID: 4Z34), S1P and (*S*)-FTY720-P entrance to the S1P_1_ receptor (refined crystal structure PDB ID: 3V2Y). We performed at least three repeats of SuMD (150–450 ns) simulations for each of these ligands.

## 4. Conclusions

Our results of SMD and SuMD simulations indicate unambiguously that the ligand binding channel is located between the extracellular parts of helices TM1 and TM7 for all three investigated lipid receptors, and it is very likely that this may be true for all the lipid receptors. The orientation of ligands during the entrance is less certain, however. The results obtained for the CB_1_ and LPA_1_ receptors indicate that ligands enter into that receptor with the “hydrophobic chain first” orientation and reach the orthosteric binding site. In the case of the S1P_1_ receptor, the more complicated mechanism appears since the agonists reach the orthosteric binding site with their very long hydrophobic tails, while the phosphate group at the other end of the ligand stays outside of the receptor binding site. However, the hydrophobic tail of agonists can reach the transmission switch, so it is probable that after the action of the switch more room is made in the S1P1 receptor interior and a deeper entrance of the agonist is possible. The mechanism of ligand entry into the binding site of lipid receptors provides important information for development of new therapeutics targeting those receptors since the way of providing a ligand to the receptor together with its binding affinity contributes to the ligand’s efficiency.

## Figures and Tables

**Table 1:** Distances between a center of mass of the ligand in the SuMD final position and the crystal ligand in its ligand-receptor crystal structure.

SuMD Ligand	Crystal Ligand	Distance [Å]
CB1 receptor ligands		
THC	7DY (PDB id: 5u09)	3.780
THC	8D0 (PDB id: 5xr8)	3.596
THC	8D3 (PDB id: 5xra)	3.595
THC	ZDG (PDB id: 5tgz)	2.745
AEA	7DY (PDB id: 5u09)	3.020
AEA	8D0 (PDB id: 5xr8)	3.211
AEA	8D3 (PDB id: 5xra)	3.463
AEA	ZDG (PDB id: 5tgz)	2.759
S1P_1_ receptor ligands		
S1P	ML5 (PDB id: 3V2Y)	7.920
S1P	ML5 (PDB id: 3V2W)	8.069
LPA_1_ receptor ligands		
(*S*)-LPA	ON3 (PDB id: 4z36)	3.346
(*S*)-LPA	ON7 (PDB id: 4z34)	3.541
(*S*)-LPA	ON9 (PDB id: 4z35)	3.556
